# Domain-Specific and Unspecific Reaction Times in Experienced Team Handball Goalkeepers and Novices

**DOI:** 10.3389/fpsyg.2016.00882

**Published:** 2016-06-21

**Authors:** Fabian Helm, Mathias Reiser, Jörn Munzert

**Affiliations:** Department of Psychology and Sport Science, Justus-Liebig-University GiessenGiessen, Germany

**Keywords:** reaction times, task specificity, perception, action, cognition, expertise, sport

## Abstract

In our everyday environments, we are constantly having to adapt our behavior to changing conditions. Hence, processing information is a fundamental cognitive activity, especially the linking together of perceptual and action processes. In this context, expertise research in the sport domain has concentrated on arguing that superior processing performance is driven by an advantage to be found in anticipatory processes (see Williams et al., 2011, for a review). This has resulted in less attention being paid to the benefits coming from basic internal perceptual-motor processing. In general, research on reaction time (RT) indicates that practicing a RT task leads to an increase in processing speed (Mowbray and Rhoades, 1959; Rabbitt and Banerji, 1989). Against this background, the present study examined whether the speed of internal processing is dependent on or independent from domain-specific motor expertise in unpredictable stimulus–response tasks and in a double stimulus–response paradigm. Thirty male participants (15 team handball goalkeepers and 15 novices) performed domain-unspecific simple or choice stimulus–response (CSR) tasks as well as CSR tasks that were domain-specific only for goalkeepers. As expected, results showed significantly faster RTs for goalkeepers on domain-specific tasks, whereas novices’ RTs were more frequently excessively long. However, differences between groups in the double stimulus-response paradigm were not significant. It is concluded that the reported expertise advantage might be due to recalling stored perceptual-motor representations for the domain-specific tasks, implying that experience with (practice of) a motor task explicitly enhances the internal processing of other related domain-specific tasks.

## Introduction

Motor actions in sports often rely on the fast reactions needed to successfully perform basic tasks such as starting a 100-meter race or defending a goal from an opponent’s attack. Two different mechanisms seem to be fundamental for what are often incredibly fast reactions. The first is basic RT as evidence for fast internal processing. This might be shorter for skilled compared to unskilled athletes. RT has been a key topic in psychological research for more than 150 years (Helmholtz, 1850; Donders, 1869/1969; see Sanders, 1998, for an overview). RT is commonly defined as a measure of time elapsing between the occurrence of a stimulus and the onset of the response to it. In the early days, Helmholtz (1850) measured RT in order to deduce the speed of peripheral conductivity, but along with Donders (1869/1969), he noticed that RT is more likely to be the time required for internal processing (see Sanders, 1998). Thus, the time taken to initiate a response can indicate the speed of this internal processing. Regarding the second mechanism, specifically for fast ball games, quick reactions may often be grounded in the advantage gained from experts having better trained anticipatory processes than novices. Anticipation implies that athletes detect critical movement features in their opponents at an early stage that allow them to predict an action outcome before the action outcome itself has been realized (Williams et al., 2002; Balser et al., 2014; Bischoff et al., 2014). This could be a reason for shorter RTs. However, the advantage of anticipatory processes does not just produce quick reactions. Such processes also offer more time to initiate a response, because the critical movement features of an opponent are recognized more precisely and at an earlier stage (Abernethy and Russell, 1987; Savelsbergh et al., 2005; Williams et al., 2011). Hence, both basic internal processing and anticipatory processes can produce quicker responses. However, they rely on distinctly different internal perceptual-motor processes.

In recent years, expertise research in the sport domain has focused mainly on the advantage of the anticipatory processes underlying superior expertise performance (Starkes, 1987; Helsen and Starkes, 1999; Williams et al., 1999, 2002; Savelsbergh et al., 2005). Although a few studies have used additional RT measurements to examine differences between skilled athletes and novices (Spirduso and Clifford, 1978; Starkes, 1987; Travassos et al., 2013), their results have been inconsistent and they did not exclude anticipatory processing in their experimental tasks such as the different results are hardly comparable. The same mixed pattern has also been demonstrated in comparisons between physically active and non-active people (Spirduso, 1980; Yandell and Spirduso, 1981; Nougier et al., 1992; O’Donovan et al., 2006). Hence, despite a comprehensive body of evidence on basic internal processing (Schmidt and Lee, 2011, for a review), less attention has been paid to the effects resulting from motor expertise in certain S–R contingencies. It should be noted that possible effects resulting from motor expertise cannot be treated separately in such S–R tasks, but tend to be combined in the sense of perceptual-motor processes. In this context, one could argue, for instance, that the quicker responses of athletes are not a general RT phenomenon (i.e., selection process), but more a result of their expertise in performing domain-specific and integrated responses (i.e., training process). According to Farrow and Abernethy (2002) and Williams et al. (2011), this expertise relies essentially on improved anticipatory perceptual components. Expertise research generally proposes that expertise effects are a result of extensive training and do not transfer to other skill domains. This notion is similar to the concept of training and transfer specificity (Thorndike and Woodworth, 1901; Magill, 2007). It predicts that motor training produces specific effects that hardly transfer to other motor skills. This issue has been demonstrated for postural control (Robertson and Elliott, 1996; Naumann et al., 2015) and for skill-relevant contextual effects (Proteau, 1992). This is in line with trainings of specific S–R contingencies for experts that rely mainly on anticipatory perceptual components (Farrow and Abernethy, 2002; Williams et al., 2011). However, any test of the effects of motor expertise on internal processing speed in an S–R task has to ensure that anticipatory perceptual processes are excluded.

Another important issue regarding internal perceptual-motor processing comes to mind when thinking about situations in which athletes have to reprogram their reactions because, for example, an opponent has performed a deceptive action. In principle, this phenomenon can be viewed as a double stimulus–response task as found in research on the PRP (Telford, 1931; Pashler, 1984; cf. Schmidt and Lee, 2011). PRP tasks contain specific S–R contingencies that may help to elucidate performance differences between various skill levels. These experimental paradigms present a close succession of two stimuli that both require a motor response. Researchers have shown that high levels of practice on these tasks reduce the dual-task costs (Gottsdanker and Stelmach, 1971; Van Selst et al., 1999). For instance, Van Selst et al. (1999) showed that the PRP effect in a speeded S–R task requiring a motor response dramatically decreased by almost 90% of the initial effect after 7 weeks of practice.

Against this background, the present study investigates the effects of motor expertise on the speed of internal perceptual-motor processing of unpredictable S–R tasks in a specific sport setting. Specifically, we ask whether simple or choice RTs are independent from or dependent on specific motor expertise—an expertise that is associated with the history of individual (training) experiences. This is a critical point for expertise research in the sport domain, because the typical expertise advantage is interpreted restrictively as anticipatory perceptual processing and not as a potentially basic internal processing (RT) advantage. Additionally, we ask whether experienced athletes (team handball goalkeepers), who can be considered to be experts in dealing with deceptive behavior, will show superior performance on a double stimulus-response task in which they have to reprogram their action. The main objectives of the present study were as follows: first, we used unpredictable simple and choice S–R tasks to study effects of motor expertise on basic internal perceptual-motor processing. This is why we examined two groups with different expertise: experienced semi to successful elite team handball goalkeepers (as classified by Swann et al., 2015) who are considered as *experts* for domain-specific responses in the form of hand or foot movements in response to a stimulus, and *novices* with no background in goalkeeping. Second, we investigated the effects of motor expertise in a double stimulus–response task that required expertise-specific motor responses, but also excluded anticipatory perceptual processes by using unpredictable stimulus onsets.

We applied a design containing a total of five experimental conditions. Participants had to use movements to react as quickly as possible to different stimuli in four basic conditions with different S–R alternatives. Participants were naïve to all stimuli used during the experiment. Movements to be made were either familiar only to the experts or they were unfamiliar to both groups. This resulted in two expertise-specific and two expertise-unspecific conditions. Specific conditions required a handball-related motor response, whereas the unspecific conditions required finger movements only. In a fifth condition, we adjusted the typical PRP paradigm to present a double stimulus–response task similar to a goalkeeper’s reaction to being deceived by an opponent. The respective responses were familiar only to the expert group. In all S–R tasks, participants could not have anticipated either the event of stimulus onset or the required motor response. We predicted that we would observe shorter RTs among the experts in comparison to the novices on those basic S–R tasks that were expertise-specific, but no differences in RTs on expertise-unspecific basic S–R tasks. This hypothesis was derived from conceptions of training and transfer specificity. For the double stimulus–response paradigm, we predicted that experts would show a significantly smaller increase in RTs for the second response than novices.

## Materials and Methods

### Participants and Design

Thirty-three male participants with normal or corrected-to-normal vision volunteered for this study (*M_age_* = 24.4 years, *SD* = 4.9). The study was approved by the local ethics committee of the Justus-Liebig University Giessen and all participants gave their informed written consent in accordance with the Declaration of Helsinki. Participants were divided into two groups: experts (semi to successful elite team handball goalkeepers, according to Swann et al., 2015, *n* = 15) and novices with experience in recreational sports, but no experience in team handball or goalkeeping (*n* = 18). Novices reported on average to exercise weekly in different sports such as (table) tennis, badminton or fitness. Team handball goalkeepers from the expert group reported practicing for an average of 8.7 h per week (*SD* = 2.5) and they had a mean playing experience of 14.3 years (*SD* = 4.4). Three participants of the novice group had to be excluded from the data analysis because they reported having an earlier history of club level experience in team handball.

In summary, we conducted an experiment with five different sessions of unpredictable S–R tasks. These tasks were designed so that participants could not anticipate either the event of stimulus onset or the required motor response in order to ensure that anticipatory perceptual processes were excluded. Stimuli figuration (symbolic pictures of a ball) in all conditions was considered to be unspecific for both the novices and the expertise domain. The responses to be made on these tasks were either a movement that was familiar only for the experts (expertise-specific) or a movement that was unfamiliar (expertise-unspecific) for both groups. A detailed description of the different experimental conditions is given below.

### Unspecific simple stimulus–response task

Participants had to release a button that they were pressing with their right or left index finger (*10 times each*) as soon as a stimulus appeared in the middle of a screen.

### Unspecific two-choice stimulus–response (2CSR) task

Participants had to release one of two buttons being pressed by their right and left index finger as soon as a stimulus appeared in the corresponding right or left half of the screen (*20 times each*).

### Specific two-choice stimulus–response (2CSR) task

Participants had to move either the left or right hand from a starting position to a target placed in the left or right upper corner of a handball goal (*20 times each*). Stimuli were the same as in the unspecific 2CSR task.

### Specific four-choice stimulus–response (4CSR) task

Participants had to move either the left or right hand from a starting position to the same targets as in the specific 2CSR task or bring together their left or right hand and foot at a specified target location in the lower left or right corner of the goal (*20 times each*). Stimuli on screen appeared in one of four quadrants.

### Specific double stimulus–response (double SR) task

**FIGURE 1:**
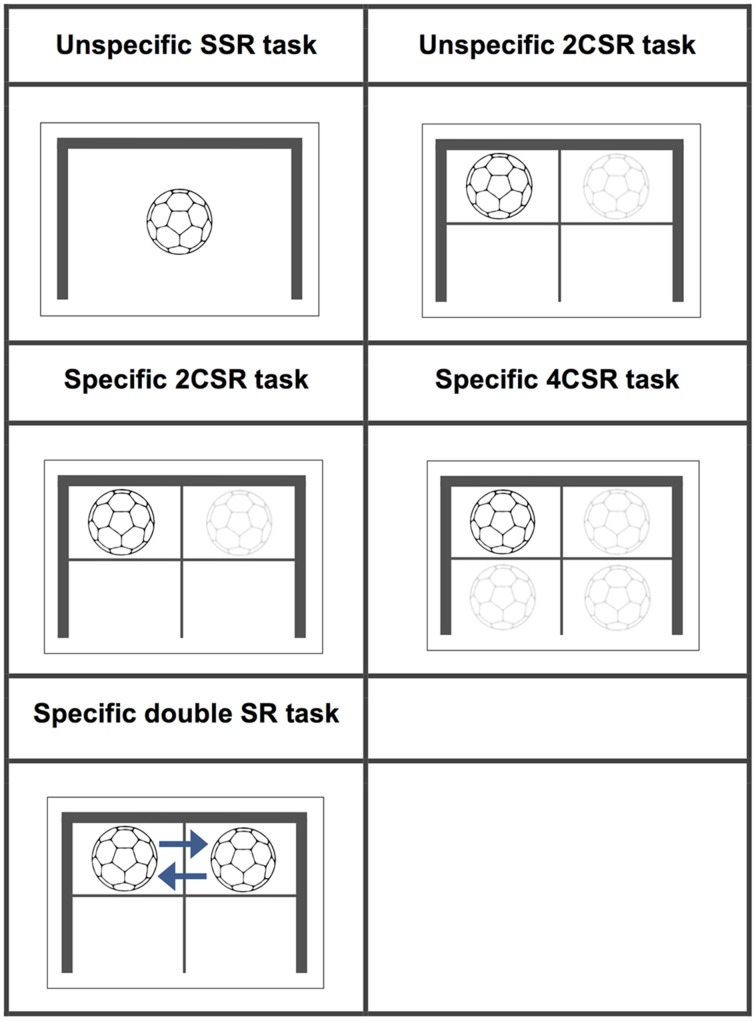
**Stimuli: characteristics for all experimental conditions**.

Participants had to react to two closely spaced stimuli (SOA: 156 ms) by moving their left followed by their right (or their right followed by their left hand) from the starting position toward the targets in the upper left or upper right corner of the handball goal (*20 times each*). They were instructed to discontinue their first response as soon as the first stimulus (S1) disappeared and the second (S2) appeared on the screen. Reaction times for the first responses are labeled double SR RT_1_, and those for the second responses are labeled double SR RT_2_. This task contained a total of 130 trials, with *40 trials* of the double SR task being embedded in a pseudo-randomized order among 90 trials of specific two-choice reactions (double SR 2CR) in which only S1 was presented. Visualizations of the stimuli for all tasks conditions together with their detailed characteristics are presented in **Figure 1**. The specific S–R tasks were considered to simulate the defensive reactions of a team handball goalkeeper and were therefore assumed to be expertise-specific to the expert group alone, whereas the unspecific tasks were expertise-unspecific for both groups. In the unspecific S–R conditions, we deliberately chose button-release tasks to compare these unspecific with the specific sports-related movement tasks. For many years, such button-press/release tasks served as a typical response type in action prediction research in sports until researchers in that domain suggested designing experiments in which participants are required to give highly domain-specific responses (Farrow and Abernethy, 2003; Mann et al., 2010).

### Procedure

Prior to the experimental block, participants attended a short test and introductory session to familiarize themselves with the experimental setting. For the experimental session, a set of six retro reflective markers was attached to their fingers, hands, and shoes. Markers were fixed directly to the skin.

During the experiment, participants stood in the middle of a handball goal in front of a small desk with a response time box placed in front of them. The screen presenting the visual stimuli (37.7 cm in the horizontal and 30.3 cm in the vertical plane) was placed 1.5 m in front of them and adjusted to each participant’s height. The specific and unspecific conditions were presented in blocks in two different sessions in a pseudo randomized order.

**FIGURE 2:**
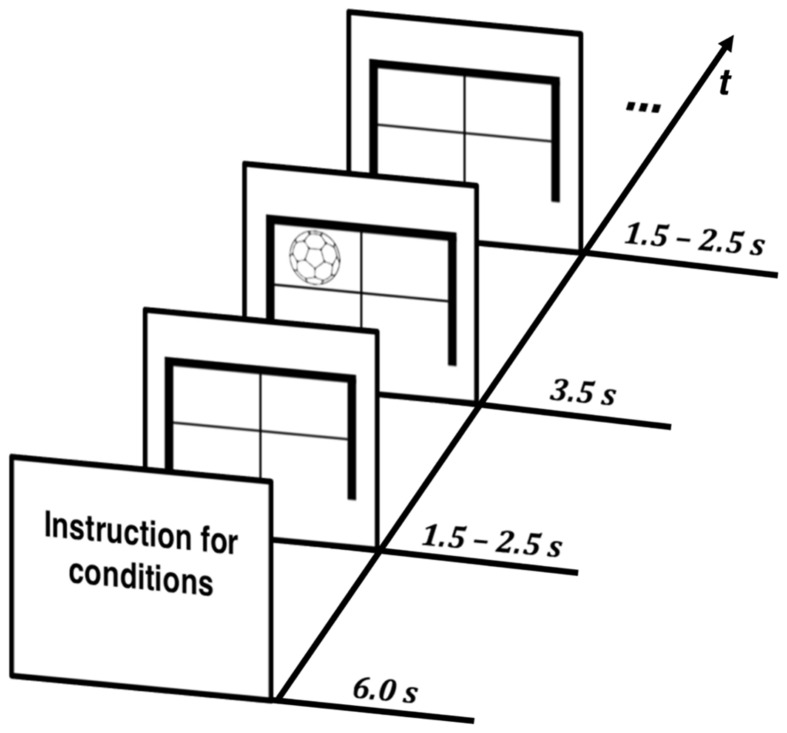
**Temporal structure: timing of stimuli for all conditions**.

Participants received a short explanation of the task in each condition and were instructed as follows: *React as quickly and accurately as possible to the corresponding stimulus and hit the targets in the specific S*–*R tasks*. All trials in each condition started with a fixation cross. This was displayed on the screen for a duration of 1.5–2.5 s before the stimulus of the corresponding task appeared. The respective time jitter was necessary to exclude anticipatory behavior by making it impossible for participants to predict the occurrence of the presented stimuli. Visual stimuli were generated at a resolution of 1280 × 1024 pixels with Presentation software (Neurobehavioral Systems, Albany, NY, USA) running on a control PC. Stimuli were presented for a duration of 3.5 s on screen in order to provide enough time for retaking the initial position after reacting to a stimulus. The timing of the stimuli for all conditions is illustrated in **Figure 2**.

### Data Collection

Movement data were collected using a motion capture system (VICON, Oxford, England) equipped with 13 CCD high speed cameras and remote controlled by the Presentation software. The system tracked three-dimensional trajectories of the retro reflective markers with a spatial accuracy of 1 mm and a temporal resolution of 240 Hz. The accuracy of RTs, calculated from the motion data, was controlled with a response time box (V5.1, LOBES, The Ohio State University, Columbus, OH, USA) guided through PsychToolbox-3 in MATLAB R2014a (MathWorks, Natick, MA, USA). The motion capture system recorded trigger signals (stimulus presentation on the screen) simultaneously with the motion data.

### Data Analysis

Motion capture data were preprocessed with Nexus 1.7 (VICON, Oxford, England). RTs in all conditions were calculated in MATLAB R2014a (MathWorks, Natick, MA, USA) as the time between stimulus and movement onset based on the velocity profiles of the markers. When calculating RTs, we took the visual delay (28 ms) when presenting the stimuli on the computer screen into account. The accuracy of motion-based RTs in the unspecific S–R tasks was controlled through the RTs measured by the response time box.

Subsequently, we inspected RTs visually and used absolute cutoffs for data correction adjusted to our experimental design (as recommended by Ramsay and Silverman, 2002, and Whelan, 2008). Additionally, we took traditional findings on RT in terms of the available number of S–R alternatives into account (cf. Schmidt and Lee, 2011). In the specific and unspecific SSR and 2CSR tasks, we discarded trials outside the interval 120 ms < RT < 450 ms (4.0%). In the specific 4CSR task, we discarded trials outside the interval 220 ms < RT < 550 ms (13.7%). Trials on the double SR task were discarded when RTs were outside the following intervals: 120 ms < double SR RT_1_ < 450 ms or 120 ms < double SR RT_2_ < 650 ms and 120 ms < double SR 2CR < 450 ms. In total, we discarded 9.0% of trials in the specific double SR condition. These cutoffs were adjusted to our task by taking account of findings on general refractoriness (cf. Schmidt and Lee, 2011). In general RT shorter than the lower boundary might be the result of fast response guesses whereas values longer than the upper boundary are the indication of inattentive response behavior (Whelan, 2008).

#### Fitting Ex-Gaussian PDF to RT Data

Because RT data generally do not have a Gaussian distribution but are more like an ex(ponential)-Gaussian distribution (Luce, 1986), that is, a convolution of two additive processes, Whelan (2008) has proposed fitting the ex-Gaussian PDF to the RT data. The ex-Gaussian PDF is described by three parameters: μ (mu), the mean of the Gaussian distributed part, σ (sigma), the standard deviation of this part, and τ (tau), the mean of the exponential part characterizing the skewness of the overall distribution (Burbeck and Luce, 1982; Lacouture and Cousineau, 2008). According to Hervey et al. (2006) and Whelan (2008) parameter μ provides the most reliable estimation of the distribution whereas the parameter τ estimates the proportion of the slower RT within the distribution. This parameter could be affected by slow RTs which are a result of inattentive participant’s behavior. On these grounds, we fitted the ex-Gaussian PDF to each participant’s RT data so that we could analyze the characteristics of whole distributions. We fitted the data with the DISTRIB-Toolbox (Lacouture and Cousineau, 2008) in MATLAB R2014a (MathWorks, Natick, MA, USA). We estimated parameters of the PDF for each participant using minimum minus LogL estimation; that is, the parameter values that were most likely given the data set. This estimation was performed with a search algorithm known as Simplex. According to Lacouture and Cousineau (2008), using the LogL criterion with the Simplex search algorithm results in the best fit of the parameters of a PDF to the data distribution.

#### Statistics

For the basic S–R tasks, we used separate 4 (condition: unspecific SSR, unspecific 2CSR, specific 2CSR, specific 4CSR) × 2 (group: experts vs. novices) ANOVAs with repeated measures for the comparison of individual differences between conditions to determine effects for mean RT and the parameters of the ex-Gaussian PDF. We conducted multiple comparison *post hoc* tests to determine the locus of significant differences for the Condition × Group interaction while controlling the family error rate with Bonferroni corrections. An additional *post hoc* 2 (condition: unspecific SSR, unspecific 2CSR) × 2 (group: experts vs. novices) ANOVA was conducted for parameter μ to validate the results of the multiple comparison *t* tests for the Condition × Group interaction.

We used separate 2 (condition: specific 2CSR, double SR RT_2_) × 2 (group: experts vs. novices) ANOVAs with repeated measures for the comparison of individual differences between conditions to determine the effects of the double SR task on mean RT and the parameters of the ex-Gaussian PDF. RTs of the specific 2CSR condition served as the control RTs. *Post hoc*, we conducted 3 (condition: specific 2CSR, double SR RT_1_, double SR 2CR) × 2 (group: experts vs. novices) ANOVAs to compare the slowdown of first RTs in trials on the RP task in which S1 was followed by S2 (double SR RT_1_) and in which no second stimulus appeared at all (double SR 2CR) with RTs in the specific 2CSR condition. This slowdown was tested for mean RT and the parameter μ of the ex-Gaussian PDF. We also conducted multiple comparison *post hoc* tests to determine the locus of significant differences while again controlling the family error rate with Bonferroni corrections.

## Results

### Basic S–R Tasks

**Table 1** reports mean RTs and the values of the ex-Gaussian PDF for RTs of the basic S–R tasks and the statistical results of the four separate 4 (condition: unspecific SSR, unspecific 2CSR, specific 2CSR, specific 4CSR) × 2 (group: experts vs. novices) ANOVAs for mean RT and the parameters μ, σ, and τ of the ex-Gaussian PDF. Referring to Whelan (2008), we shall focus on the results of the normal distributed portion of RTs, parameter μ. This parameter provides the most reliable estimation of the distribution. The important effects for this parameter are shown by a significant Condition × Group interaction, *F*(2.21,61.95) = 4.55, *p* = 0.012, $\eta_{p}^{2}$ = 0.14. This interaction was also significant for the parameter σ, *F*(2.31,64.7) = 4.27, *p* = 0.014, $\eta_{p}^{2}$ = 0.13. *Post hoc* multiple comparisons for parameter μ revealed significant effects of only shorter RTs for the experts on the specific 2CSR (experts vs. novices: *t*[28] = 3.37, *p* < 0.01) and the specific 4CSR condition (experts vs. novices: *t*[28] = 3.26, *p* < 0.01) task, but not for the unspecific SSR, *t*(28) = 1.60, *p* = 0.12, and the unspecific 2CSR condition, *t*(28) = 1.00, *p* = 0.32. An additional *post hoc* 2 (condition: unspecific SSR, unspecific 2CSR) × 2 (group: experts vs. novices) ANOVA for parameter μ revealed a significant effect for condition, *F*(1,28) = 42.84, *p* < 0.001, $\eta_{p}^{2}$ = 0.61; but not for group, *F*(1,28) = 1.92, *p* = 0.18, $\eta_{p}^{2}$ = 0.064; and not for the Condition × Group interaction, *F*(1,28) = 0.019, *p* = 0.89, $\eta_{p}^{2}$ = 0.001.

**Table 1:** Average result pattern for basic S–R tasks: mean reaction times, parameters from fitting the ex-Gaussian PDF, and statistical results of within-subject and between-group effects.

	Experts, *n* = 15				Novices, *n* = 15						
	SSR unspecific	2CSR unspecific	2CSR specific	4CSR specific	SSR unspecific	2CSR unspecific	2CSR specific	4CSR specific	Condition *F*	Group *F*	Condition × Group *F*
Mean RT	192.7 (20.3)	228.7 (24.0)	218.4 (21.0)	280.5 (25.6)	213.3 (25.7)	250.0 (28.6)	258.9 (31.0)	321.2 (29.6)	132.73^∗∗∗^	18.0^∗∗∗^	2.56
μ (mu)^1^	170.0 (18.0)	196.6 (20.7)	193.8 (24.6)	242.6 (21.0)	181.1 (20.0)	206.6 (32.6)	232.8 (37.5)	279.1 (37.9)	93.59^∗∗∗^	9.58^∗∗^	4.55^∗^
σ (sigma)^1^	11.4 (6.9)	20.1 (8.4)	16.3 (8.6)	14.8 (12.7)	11.2 (8.0)	19.6 (9.7)	20.2 (8.2)	29.8 (14.8)	7.16^∗∗^	5.17^∗^	4.27^∗^
τ (tau)^1^	22.6 (13.1)	32.1 (14.5)	24.6 (11.3)	37.9 (12.1)	32.2 (15.9)	43.4 (19.6)	26.0 (18.7)	42.1 (12.8)	11.43^∗∗∗^	2.74	1.13

^1^*Parameters from fitting the ex-Gaussian distribution to the data. All values are group means in ms, values in parentheses indicate SD. ^∗^p < 0.05, ^∗∗^p < 0.01, ^∗∗∗^p < 0.001.*

**FIGURE 3:**
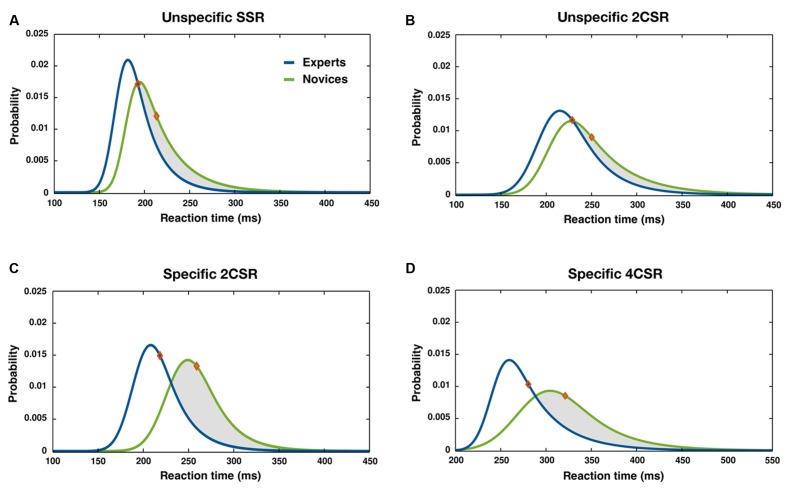
**Distribution of RT for specific and unspecific basic S–R tasks: ex-Gaussian PDFs and mean RTs (diamonds) separated by groups for unspecific SSR (A), unspecific 2CSR (B), specific 2CSR (C), and specific 4CSR (D) tasks**. Intervals of RT cutoff: **(A–C)**: 120 ms < RT < 450 ms, **(D)**: 220 ms < RT < 550 ms.

Significant group effects emerged for parameter μ and also for parameter σ, with shorter RTs for the expert group (see **Table 1**). These effects together with the significant Condition × Group interaction and its subsequent *post hoc* test results demonstrated that group differences resulted mainly from the two- and four-choice expertise-specific S–R tasks. **Figure 3**, illustrating the RT distributions for conditions by groups, shows that the distributions for both groups were rather similar for the unspecific S–R tasks but differed for the expertise-specific tasks. In the latter case, the distributions for the novices shifted more to the right, indicating a higher proportion of excessively slow reactions. It was conspicuous that such a skewness could not be found for RTs in the unspecific SSR and particularly not in the unspecific 2CSR task.

### Double Stimulus–Response Task

**FIGURE 4:**
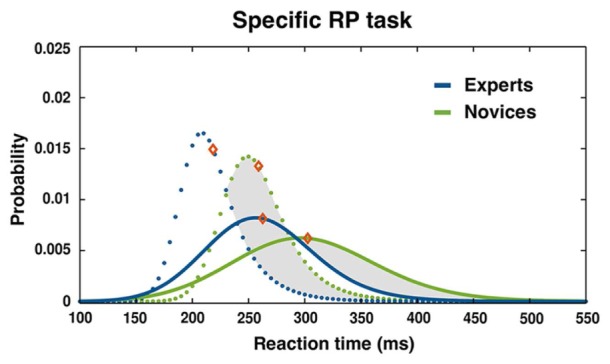
**Distribution of RT for specific double SR task: ex-Gaussian PDF and mean RTs (diamonds) for RT_1_ (dashed lines) and RT_2_ (solid lines) separated by groups.** Intervals of RT cutoff: 120 ms < RT_1_ < 450 ms, 120 ms < RT_2_ < 650 ms.

The four separate 2 (condition: specific 2CSR, double SR RT_2_) × 2 (group: experts vs. novices) ANOVAs revealed a significant effect of condition for the parameter μ, *F*(1,28) = 5.11, *p* = 0.032, $\eta_{p}^{2}$ = 0.15; and σ, *F*(1,28) = 20.18, *p* < 0.001, $\eta_{p}^{2}$ = 0.42, of the ex-Gaussian PDF with longer RTs and a highly increased variance of distributions for the second RT (RT_2_) in comparison to the specific 2CSR condition. This clearly revealed the typical effects of PRP tasks. Additionally, we found a significant effect of group for parameter μ, *F*(1,28) = 4.23, *p* < 0.05, $\eta_{p}^{2}$ = 0.13, with generally shorter RTs for the experts, thereby underlining the general RT advantage of the expert group in specific S–R tasks. There were no significant effects for the Condition × Group interaction (see **Table 2**). The ex-Gaussian PDFs by groups are illustrated in **Figure 4**, and average values with statistical results are reported in **Table 2**. *Post hoc* 3 (condition: specific 2CSR, double SR RT_1_, double SR RP 2CR) × 2 (group: experts vs. novices) ANOVAs to control for the RT slowdown of all first responses on the double SR task (double SR RT_1_, double SR 2CR) in comparison with the RTs of the specific 2CSR condition showed significant effects of condition (μ: *F*[2,56] = 28.82, *p* < 0.001, $\eta_{p}^{2}$ = 0.51) and group (μ: *F*[1,28] = 9.45, *p* = 0.005, $\eta_{p}^{2}$ = 0.25) with slower RTs for the novices, but no Condition × Group interaction (see **Table 3**). *Post hoc* multiple comparisons for parameter μ for conditions revealed a significant effects for the comparison double SR RT_1_ versus 2CSR, *t*(29) = 6.5, *p* < 0.001, with slower reactions for the first responses on the double SR task, but not for double SR 2CR versus 2CSR. Additionally, we found a significant effect for the comparison between the first responses in which S1 was followed by S2 and in which no second stimulus was presented at all (double SR RT_1_ vs. double SR 2CR: *t*[29] = 6.78, *p* < 0.001). Surprisingly, participants showed slower reactions when the first response was followed by a second. **Table 3** reports the results of the repeated measures ANOVAs and the average values for RT slowdown.

**Table 2:** Average result pattern for double SR task: mean reaction times, parameters from fitting the ex-Gaussian PDF, and statistical results of within-subject and between-group effects.

	Experts, *n* = 15		Novices, *n* = 15				
	2CSR specific	Double SR RT2	2CSR specific	Double SR RT2	Condition *F*	Group *F*	Condition × Group *F*
Mean RT	218.4 (21.0)	262.6 (69.0)	258.9 (31.0)	302.6 (82.8)	10.40^∗∗^	6.53^∗^	0.00
μ (mu)^1^	193.8 (24.6)	233.4 (77.3)	232.8 (37.5)	267.7 (98.6)	5.11^∗^	4.23^∗^	0.02
σ (sigma)^1^	16.3 (8.6)	41.8 (25.8)	20.2 (8.2)	56.4 (45.4)	20.18^∗∗∗^	1.75	0.61
τ (tau)^1^	24.6 (11.3)	29.2 (23.6)	26.0 (18.7)	34.9 (39.5)	0.99	0.31	0.10

^1^*Parameters from fitting the ex-Gaussian distribution to the data. All values are group means in ms, values in parentheses indicate SD. ^∗^p < 0.05, ^∗∗^p < 0.01, ^∗∗∗^p < 0.001.*

**Table 3:** Slowdown of reactions (RT_1_, 2CR) in the specific Double SR task: average values and statistical results of the comparison with the specific 2CSR task.

	Experts, *n* = 15			Novices, *n* = 15					
	2CSR specific	Double SR RT1	Double SR 2CR	2CSR specific	Double SR RT1	Double SR 2CR	Condition *F*	Group *F*	Condition × Group *F*
Mean RT	218.4 (21.0)	266.2 (35.5)	244.3 (30.8)	258.9 (31.0)	292.1 (33.8)	271.6 (27.7)	31.96^∗∗∗^	11.03^∗∗^	1.26
μ (mu)^1^	193.8 (24.6)	240.7 (43.5)	209.4 (37.6)	232.8 (37.5)	276.1 (37.9)	239.1 (35.8)	28.82^∗∗∗^	9.45^∗∗^	0.29

^1^*Parameters from fitting the ex-Gaussian distribution to the data. All values are group means in ms, values in parentheses indicate SD. ^∗^p < 0.05, ^∗∗^p < 0.01, ^∗∗∗^p < 0.001.*

## Discussion

The present study used expertise-specific and unspecific S–R tasks to investigate the effects of motor expertise on the speed of internal perceptual-motor processing. The main goals were twofold: first, we wanted to investigate whether motor-experienced athletes (team handball goalkeepers) would show a superior performance in basic perceptual-motor processing; and second, we wanted to identify whether the predicted expertise advantage would be due only to the processing of domain-specific movements or would be a general advantage. By examining the whole RT distribution, we were able to perform a detailed and comprehensive analysis and detect effects that would otherwise have been missed. In general, our results replicate early findings showing an increase in RTs associated with an increase in S–R alternatives (Hick, 1952; Hyman, 1953). Our main results are as follows: first, experts tend to show, on average, quicker reactions than novices. Second, experts specifically show a significant advantage on domain-specific S–R tasks, whereas novices tend more frequently to produce excessively long RTs on such tasks. Third, experts and novices show different RTs in a double-response paradigm. The following sections will discuss these results in detail.

### Effects of Motor Expertise on Basic Internal Perceptual-Motor Processing

One often reported assumption regarding expertise research in sports is that the production of fast reaction times by skilled athletes is grounded mainly in an advantage of anticipatory perceptual processes rather than in an efficiency of basic perceptual-motor processing for domain-specific movement tasks resulting from the expertise motor experience. Within this context, our results demonstrate that it is not exclusively superior anticipatory performance, but especially an efficiency of perceptual-motor processing on domain-specific movement tasks that induces fast reactions in skilled (motor experienced) athletes. Our results also support findings on action prediction research in the sports domain, showing that the expertise advantage increases when athletes are required to perform specific sports-related reactions during more natural paradigms (Farrow and Abernethy, 2003; Mann et al., 2010; Travassos et al., 2013).

Up to now, only a few studies have shown a reduction in averaged RTs and RT variability as a result of practice (Mowbray and Rhoades, 1959; Rabbitt and Banerji, 1989). In relation to our study design, we note explicitly that our expertise-specific response tasks are classified as being similar to a typical defensive reaction by a team handball goalkeeper. This implies that our specific S–R tasks have not been practiced in their task-specific manner by one group or the other. It is only the practice of a similar domain-specific reaction (the goalkeeper’s save) that is taken into account for the expertise of the goalkeeper, because the experimental tasks differed only across the required movement responses, but not with respect to the figuration of presented stimuli. Taking all this together, we suggest that practicing perceptual-motor tasks enhances the processing of other related domain-specific S–R tasks which require different movement responses. The fact that the expert group shows less variation on the specific S–R tasks supports this line of reasoning. One central assumption of (sensory motor) learning theory and expertise performance is that practice produces an acquired capability for skilled movements that generates a “storage” of refined internal (sensory motor) representations (Ericsson and Smith, 1991; Beilock et al., 2003; Ericsson, 2003, 2007; Frank et al., 2013). These circumstances could either bypass or inherently alter the basic limits of internal processing through training (Ericsson, 2003). We argue that experienced goalkeepers establish these sensory motor representations while performing domain-specific reactions over their years of training. We suggest that recalling these stored representations facilitates internal perceptual-motor processing; and that it was this that resulted in faster RTs in our expertise-specific S–R tasks. This indicates that domain-specific (perceptual-motor) training facilitates not only anticipatory perceptual processes (Farrow and Abernethy, 2002; Williams et al., 2011) but also, and especially, internal perceptual-motor processing. The effects might become even stronger with an increased amount of practice or movement experiences over the life span as reported in expertise research (Ericsson et al., 1993). That this strong facilitation does not hold for the unspecific S–R tasks demonstrates the aforementioned notion that perceptual-motor training produces specific effects that do hardly transfer to other skills. The goalkeepers’ perceptual-motor expertise does not facilitate the internal processing of domain-unspecific S–R tasks. In this context, we cannot totally rule out that quicker responses by athletes are not a general RT phenomenon (i.e., selection process), but our data indicate that a stronger facilitation of internal processing might be a result of perceptual-motor expertise in performing domain-specific and integrated responses (i.e., training process). However, the RT distributions in the unspecific S–R tasks already indicate that novices tend more frequently to produce long RTs than experts do. Indeed, although differences are not statistically significant, the possibility that early selection processes lead to only athletes with better internal processing abilities remaining in the goalkeeping domain cannot be precluded.

Taking traditional information processing models into account (cf. Donders, 1869/1969; Sternberg, 1969), we propose that the efficiency of internal processing in the expertise-specific tasks is driven by a more efficient response processing stage. By separating the processing stages of such simplified models into the stages of stimulus (perceptual) and response (motor) processing, we argue that our specific and unspecific 2CSR tasks differ only in terms of different motor responses to be made as a reaction to the same stimuli. Our results revealing no significant differences in RTs between groups in the unspecific but significant differences in the specific 2CSR tasks and therefore indicate an efficiency of response (motor) processing in experts.

### Double Stimulus–Response Task

As stated above, we assumed that our double-response task would be grounded in the same mechanisms as those described for the PRP paradigm. In this context, our results reveal a similar pattern to early findings reported by Welford (1968) showing an increase of RTs for the second reaction in comparison to an analogous choice reaction time in which only one stimulus is presented. However, the present significant effect of superior expertise for performance on domain-specific basic S–R tasks does not hold for the double-response task. This is in contrast to findings showing that dual-task costs decrease with the level of practice (Gottsdanker and Stelmach, 1971; Van Selst et al., 1999), indicating first and foremost that experienced team handball goalkeepers do not benefit from their internal representations when performing this task. On further consideration, we suggest that the missing effect could imply that goalkeepers do not reprogram their actions in a real situation. We propose two different lines of reasoning: first, goalkeepers tend to react to all types of throws regardless of whether they are deceptive or non-deceptive. Additionally, Cañal-Bruland and Schmidt (2009) have shown that team handball goalkeepers are biased to view a 7-meter throw as deceptive. These results could support our findings showing a slowdown of RT for the first responses on all trials of the double SR task, even though all of these responses that the participants had to make were embedded in the context of a 2CSR task. What is surprising is the effect that RT_1_ was, on average, slower than the reactions on the 2CR trials. This signifies that participants especially show a longer processing of the first reactions when S1 is followed by S2. *A priori*, we predicted only the reversed interference effect. We would argue that because S2 occurs at such an early point in time during the processing of S1, it leads to a delay in processing. Findings in the context of cost–benefit analysis for the anticipation of actions might support these effects. Several studies have shown that the inhibition of an already planned action requires more processing time considered as the cost of (re)acting incorrectly (Schmidt and Gordon, 1977; Posner et al., 1978). In general, this phenomenon could have exerted a decisive influence on the nature of internal processing in the double SR task, and could have eliminated significant differences in processing times between groups. It seems that the emergence of S2 at this point in time influences the processing of S1, and this eliminates the potential efficiency of recalling internal representations.

The second line of reasoning focuses on the nature of interactions between a goalkeeper and a field player in a real 7-m situation. Considering the time window in which goalkeepers can recognize a deceptive movement and the start of an ongoing throw, goalkeepers might have enough time to process each event separately. This implies that they have enough time to finish reacting to the field player’s first movement, to move back to their initial position, and to start a possible second response. Consequently, a typical reprogramming of movements may well be unnecessary in the majority of real-life 7-m situations. This is why the goalkeepers’ domain-specific perceptual-motor expertise will not help to facilitate the internal processing of the double-response task, and we consider *post hoc* that this task is probably unspecific in the goalkeeper’s domain. However, we deliberately chose an SOA of 156 ms in our paradigm to force participants not to process the two stimuli either grouped together or one after the other, respectively not independently. Due to both a goalkeeper’s predisposition to judge actions as deceptive and the short time interval between the first and the second stimuli, goalkeepers might have deliberately slowed down their first reaction, and this would account for the lack of any effect of RT differences between experts and novices in the second reaction. The effect of slowdown in the novice group could be explained through cost–benefit trade-offs (cf. Schmidt and Gordon, 1977; Posner et al., 1978). We would suggest that the costs of being deceived on a double SR trial would be reduced by a (general) RT slowdown, and that this would facilitate correct responses.

## Conclusion

The present data reveal that motor expertise with its associated internal representations explicitly facilitates the perceptual-motor processing of domain-specific S–R tasks. Mowbray and Rhoades (1959) have already shown that practice of a traditional RT task increases the speed of internal processing. Our data extend this by showing these effects of efficiency for the processing of S–R tasks in the context of an expertise domain specificity. Due to the elimination of anticipatory perceptual behavior in the experimental tasks, this efficiency can be seen to result from expertise based on motor experience. The contrasting findings between our domain-specific and domain-unspecific S–R tasks indicate that the goalkeepers’ perceptual-motor expertise is beneficial in other tasks only within their specific perceptual-motor domain. Nonetheless, we cannot rule out the possibility that this expertise (team handball goalkeeping) leads to a general advantage in the processing of S–R tasks.

The data also reveal that the internal processing of a second stimulus that closely follows a first stimulus generally takes longer in comparison to a task in which only one stimulus is presented. In particular, our data indicate that this delay in processing affects the processing of not only the second but also the first reaction—as indicated by slower reactions in comparison to a control task. We conclude that behavioral effects of a cost–benefit trade-off influence the internal perceptual-motor processing in a real-world double stimulus-response task in general. However, our double-response task shows that further research needs to explore the nature of movement reprogramming for skilled sports performance in real-world situations.

Turning to applied contexts, we emphasize that fast reactions of athletes are not grounded exclusively in an advantage regarding action prediction, but especially in an advantage regarding internal perceptual-motor processing.

## Author Contributions

FH: designed and performed the experiment, the analysis; and wrote the manuscript. MR: contributed to all parts of this research. JM: contributed to all parts of this research.

## Conflict of Interest Statement

The authors declare that the research was conducted in the absence of any commercial or financial relationships that could be construed as a potential conflict of interest.

## Abbreviations

ANOVA: analysis of variance
CSR: choice stimulus–response
LogL: log-likelihood
PDF: probability density function
PRP: psychological refractory period
RT: reaction time
RT_1_: first reaction time
RT_2_: second reaction time
S1: first stimulus
S2: second stimulus
S–R: stimulus–response
SOA: stimulus onset asynchrony
SSR: simple stimulus–response
